# Leveraging digital transformation for better health in Europe

**DOI:** 10.2471/BLT.22.289132

**Published:** 2022-12-01

**Authors:** Hans Henri P Kluge, Natasha Azzopardi-Muscat, David Novillo-Ortiz

**Affiliations:** a World Health Organization, Regional Office for Europe, Marmorvej 51, 2100 København, Denmark.

The coronavirus disease 2019 (COVID-19) pandemic has heavily affected the Member States of the World Health Organization (WHO) European Region. WHO’s goals of advancing universal health coverage, protecting people in emergencies, and enhancing health and well-being have been challenged and delayed. However, these challenges can be addressed by leveraging the full potential of digital solutions to enhance health systems and services and reduce health inequalities. Therefore, in 2020 the WHO European Region made digital health one of the four flagship areas of the European Programme of Work,^1^ a decision agreed to by all 53 Member States of the Region. Furthermore, in 2022, the Regional Digital Health Action Plan 2023–2030 gives us a roadmap to achieve these goals; the plan has been consulted with and adopted by all Member States and relevant partners.^2^^,^^3^

The pandemic has further underscored the importance of health information and sparked innovative ways of collecting and processing data. Health information systems have been strained from the onset of the pandemic, highlighting the need to implement digital solutions that improve the efficiency of these systems.

The WHO European Region has been facing many challenges even before the pandemic started. At the country level, decision-makers have different understandings of what digital health means, and long-term vision of the needs, including regular allocated funding, is often lacking. Digital health should be seen as a genuine enabler to achieve health goals, not as the solution for health problems. Without governance, legislation and policies, implementing digital solutions will not be possible. For example, digitalizing data and information systems is insufficient; if the data process is broken, its digitalization will continue to be broken. The absence of data to facilitate informed decisions and investments around digital health is another obstacle, as is insufficient involvement by final users, including citizens and health workers. At the regional level, the main challenge is the lack of a common direction so that countries, according to their capacities and resources, can work together, leaving no one behind.

In response to these challenges, in this editorial we present our views and the actions needed, reflected in the Regional Digital Health Action Plan 2023–2030 and in line with the Global Digital Health Strategy,^4^ to set a regional direction. We will support countries in leveraging and scaling up their digital transformation for better health and in line with their health needs, while fully respecting the values of equity, solidarity and human rights. Some of this work has already begun. For example, in the Central Asian Republics (Kazakhstan, Kyrgyzstan, Tajikistan, Turkmenistan and Uzbekistan), Georgia, the Republic of Moldova and Romania, we are supporting authorities in strengthening health information systems to enhance health system response and establishing telemedicine services in rural areas to mitigate disruption of services and deliver high-quality primary health care.

Digital health will play an important role in the future of health and well-being, but we need to ensure that this ongoing transformation is guided by principles that reflect current challenges. We also need to ensure that expectations on how digital solutions can affect our lives and health systems become a reality.

The guiding principles of the regional digital health action plan include: (i) placing the individual at the centre of trustworthy care delivered digitally; (ii) understanding health systems’ challenges, including health needs and trends, and acknowledging the needs and expectations of citizens and health workers; (iii) recognizing the need for policy decision-making based on data, evidence and lessons learnt; (iv) leveraging digital transformation to reimagine the future of health systems; and (v) recognizing that institutionalization of digital health requires a long-term commitment and an integrated care approach.

With these principles in mind, we propose four strategic priorities to leverage digital transformation for better health in the WHO European Region. First, and considering national health systems’ needs and health priorities, we will continue setting norms, developing evidence-based technical guidance, and formulating direction to support decision-making and increased capacity in digital health.

Second, we will enhance country capacities to better govern digital transformations in health while advancing digital literacy to help achieve national health goals, improve health system performance, and guide future digital health investments.

Third, we will continue building networks and promoting dialogue and knowledge exchange to facilitate interaction between partners, stakeholders and the wider public to steer the agenda for innovation in digital health.

Finally, we will conduct horizon scanning and landscape analysis to identify patient-centred digital solutions, scaling them up at the country and regional levels.

Countries can count on the support of WHO to help them strengthen their pathways for the digital transformation of health systems. Together with partner organizations and in alignment with global efforts to standardize digital health adoption, we will promote access to digital health services among the vulnerable populations in European societies. Working together, these four goals can be achieved.
